# Conduction in the Heart Wall: Helicoidal Fibers Minimize Diffusion Bias

**DOI:** 10.1038/s41598-018-25334-7

**Published:** 2018-05-08

**Authors:** Tristan Aumentado-Armstrong, Amir Kadivar, Peter Savadjiev, Steven W. Zucker, Kaleem Siddiqi

**Affiliations:** 1 School of Computer Science and Center for Intelligent Machines, McGill University, Montréal, Canada; 2 Department of Diagnostic Radiology, McGill University, Montréal, Canada; 3 Department of Computer Science and Department of Biomedical Engineering, Yale University, New Haven, USA; 4 Department of Mathematics and Statistics, McGill University, Montréal, Canada

## Abstract

The mammalian heart must function as an efficient pump while simultaneously conducting electrical signals to drive the contraction process. In the ventricles, electrical activation begins at the insertion points of the Purkinje network in the endocardium. How does the diffusion component of the subsequent excitation wave propagate from the endocardium in a healthy heart wall without creating directional biases? We show that this is a consequence of the particular geometric organization of myocytes in the heart wall. Using a generalized helicoid to model fiber orientation, we treat the myocardium as a curved space via Riemannian geometry, and then use stochastic calculus to model local signal diffusion. Our analysis shows that the helicoidal arrangement of myocytes minimizes the directional biases that could lead to aberrant propagation, thereby explaining how electrophysiological principles are consistent with local measurements of cardiac fiber geometry. We discuss our results in the context of the need to balance electrical and mechanical requirements for heart function.

## Introduction

The heart can be viewed as a mechanical device or as an electrical device, and these must work in concert for billions of pumping contractions over an average lifetime. During every beat, the cardiac fiber geometry must support safe propagation of the electrophysiological contraction signal, or irregular patterns in the conduction wave could lead to potentially deadly arrhythmias^1^. In the ventricular system, the earliest activation sites are associated with the entry points of the Purkinje network in the sub-endocardium^2^. In the present article, our aim is to study how the excitation wave propagates from these sites without introducing directional biases in its diffusion component. The answer, we shall show, lies in the helicoidal arrangement of heart wall myofibers.

The heart is largely composed of locally parallel bundles of contractile cardiac muscle cells embedded in an extracellular matrix^3^. Analysis methods from differential geometry have shown that the orientations of these fibers approximate a minimal surface in ${{\mathbb{R}}}^{4}$, the generalized helicoid model (GHM)^4^. Such helicoidal fiber geometry affords great mechanical strength^5^, as the club-like appendages of the mantis shrimp^6^ and the tough dermal armor of certain fish^7^ illustrate; indeed, the enhanced damage tolerance is leading to applications in material science as well^8,9^.

The organization of the cardiomyocytes (i.e. their local orientation) determines a number of essential properties of the heart, including the diffusive propagation of the contraction signal and mechanical efficiency^10^. Several studies have postulated mechanistic reasons for the local helicoidal geometry (e.g.^11,12^) and, from an evolutionary perspective, it seems to emerge with the need for high blood pressure in mammals and birds^13^. Mechanical considerations alone do not suffice, however; cardiac tissue is unique in that mechanical resilience must coexist with active contraction and signal propagation.

Electrophysiologically, the cardiomyocyte fibers exert a powerful local effect on the rate of signal propagation: diffusion within fibers (and between them via end-to-end connections), is much faster than in directions orthogonal to the fibers^14–16^. Based on this, heart tissue has been modeled as a Riemannian manifold with the metric tensor determined by the local fiber direction. An important result^17^, which we confirm, is that the tissue can be approximated by a manifold with negative scalar curvature. Mathematically, this enhances diffusion in abstract manifolds^18,19^ and in the walls of the ventricles^17^; it also plays a role in models of molecular diffusion^20,21^.

In more detail, the effect of the cardiac fiber architecture on diffusion of the contraction signal has important physiological and biomedical implications. Disturbances in the signal propagation can lead to ventricular fibrillation and death, and the transmural rotation of the fibers can lead to wave breakup and chaotic behavior of wave filaments. Taken together, this implies that the fiber geometry could be a potentially dangerous source of destabilization^22–26^. How the heart wall fiber geometry balances the competing mechanical need for efficient contraction, with the electrophysiological requirement that irregular wave patterns be avoided, is an open question.

**Figure 1:**
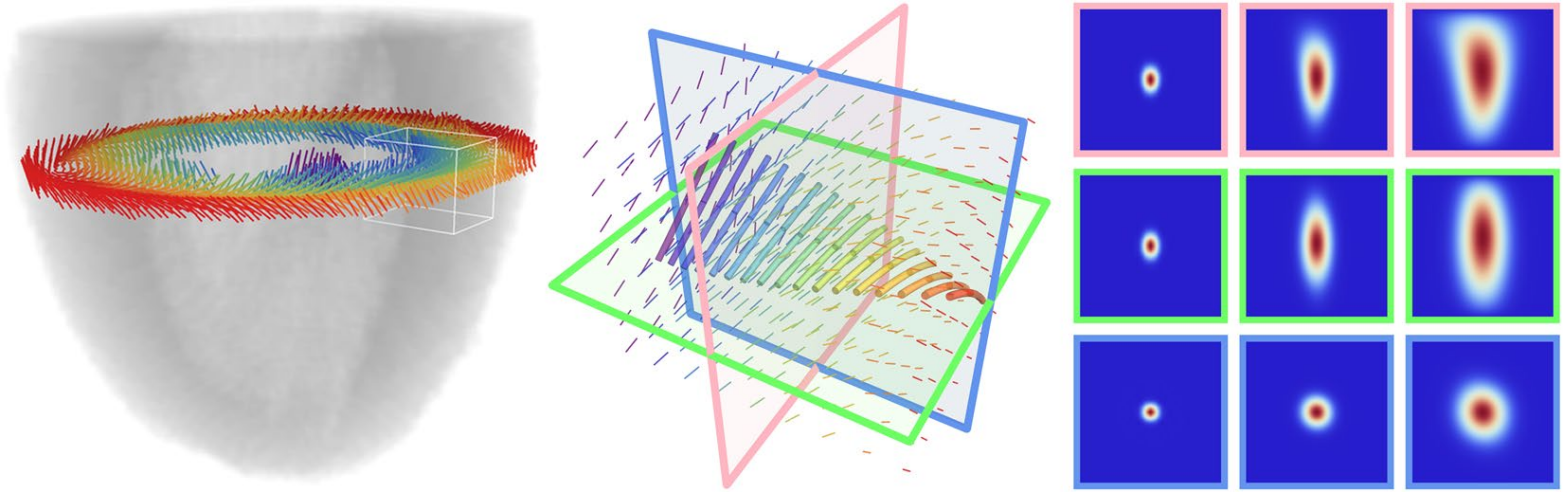
Left: a rat left ventricle, with a slice of fiber data from diffusion MRI (color denotes transmural position). Middle: displays the fiber field (from the white cube of the left inset), as well as a local GHM fit (shown as the larger, thicker curves). Right: shows the diffusion behavior in the planes of corresponding border color from the central inset (where columns represent times *t* = 0.02, 0.3, 1.0) using the GHM parameters from the local fit (see Fig. 5 for further details).

**Figure 2:**
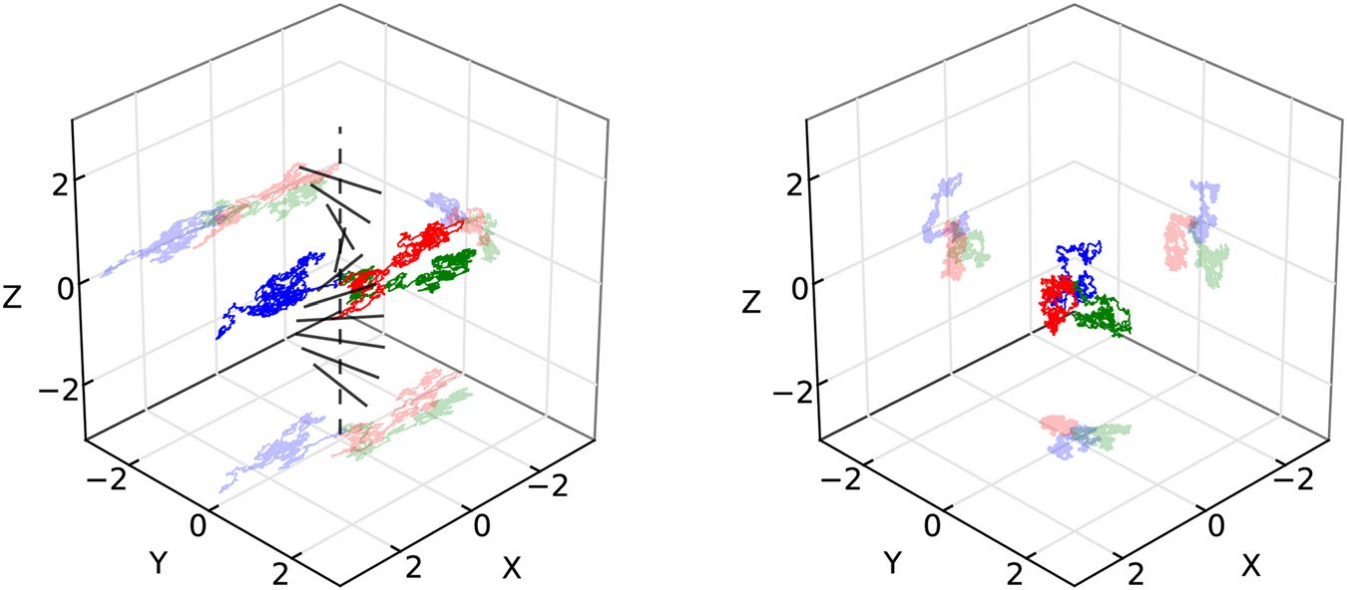
Three examples of Brownian motions (BMs) in the GHM manifold, under various speed settings, for *t* = 0 to 0.5 (with 1200 integration steps) with *k*_*B*_ = 0.9 and *k*_*N*_ = *k*_*T*_ = 0. Projections of each trajectory are shown on the axial planes in lighter shades. Simulation was computed via an order 1.0 Stochastic Runge-Kutta algorithm^54,55^. Left: *v*_*f*_ = 3, *v*_*t*_ = 1. Right: *v*_*f*_ = 1, *v*_*t*_ = 1. The line segments on the left represent the local fiber directions along the *z*-axis. Notice that the anisotropic case (left) leads to spatially skewed diffusion, in comparison with the behavior of the isotropic case (right). This is particularly noticeable along the *x* axis, which coincides with the fiber orientation at the origin, where each trajectory begins.

Classically, the propagation of the excitation signal is described by a reaction-diffusion equation, where the diffusion component is anisotropic and spatially varying^27,28^. Because we are interested in the local behavior around the insertion points of the Purkinje network in the endocardium, we focus on the diffusion term and introduce the effect of local fiber geometry via a GHM (see Fig. 1). The duality between the diffusion equation and Brownian motion (BM) allows us to move beyond the average ensemble behavior to individual sample functions and their variance (see Fig. 2). We analyze the properties of BM as an Ito process on the cardiac manifold, and our key results are that (i) there is an acceleration effect on the diffusion process due to the GHM structure, (ii) the GHM structure minimizes local directional biases in the diffusion component of the conduction wave, and (iii) within the heart wall tangent plane, the stochastic component of diffusion, i.e., its variance, becomes isotropic very quickly. All three results require that there be transmural rotation of fiber orientation in the heart wall. In the end, then, the helicoidal fiber geometry supports efficient mechanical operation^11,29^ while also reducing directional biases in electrical signal propagation.

## Model

### The Generalized Helicoid Model

To first approximation, the fiber geometry in the left ventricle has long been empirically known to show a transmural rotation through the wall, essentially irrespective of position^30,31^. This forms the basis of rule-based models of the ventricular fiber structure as well^32^. To model this, one can define the *z*-axis to lie along the transmural direction, and define an orientation (or fiber angle) function *θ*(*x*, *y*, *z*), which describes the angle of rotation of the local myofiber at the given position.

**Figure 3:**
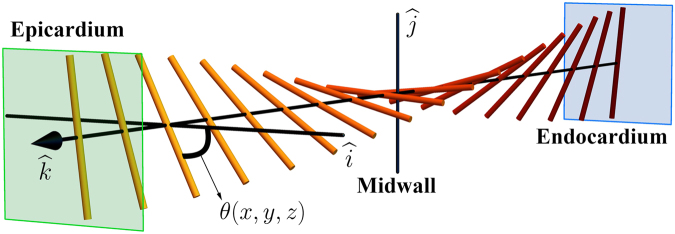
An illustration of an exemplar GHM, including a depiction of the local transmural direction (i.e. the *z*-axis, parallel to the unit vector $\hat{k}$), as well as the meaning of the local *θ* value, determined by the GHM orientation function. In this example *k*_*T*_ = *k*_*N*_ = 0.

**Figure 4:**
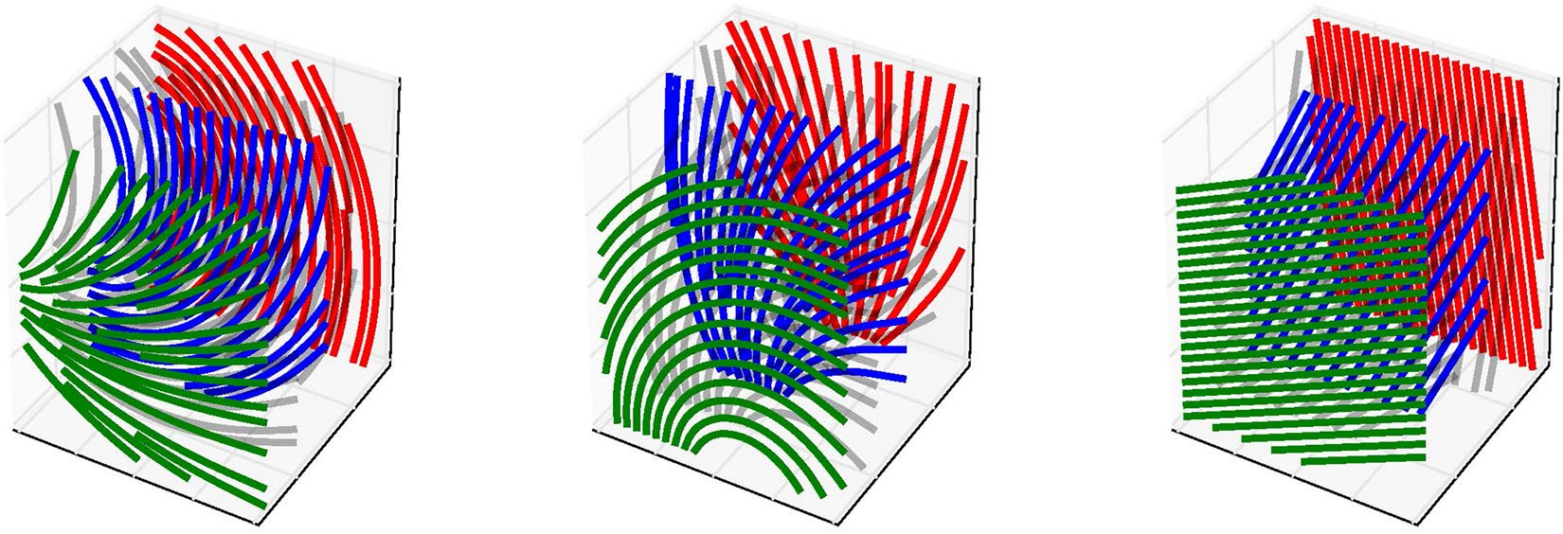
Illustration of GHM parameter values via streamline plots. Parameters (*k*_*N*_, *k*_*T*_, *k*_*B*_) for each column are given left to right as (0.02, 0, 0.7), (0, −0.02, 0.7), and (0, 0, 0.7). Planes (green, red, and blue) are at fixed points on the *z*-axis (i.e. normal to the transmural direction *z*, in the heart). Notice the in-plane fanning and bending effects, respectively, of the in-plane curvatures *k*_*N*_ and *k*_*T*_, and the transmural turning induced by *k*_*B*_.

The full expression for the orientation function of the GHM may be written as^4^:

1 $$\theta (x,y,z)=\arctan (\frac{{k}_{T}x+{k}_{N}y}{1+{k}_{N}x-{k}_{T}y})+{k}_{B}z$$

where *k*_*B*_, *k*_*T*_, ${k}_{N}\in {\mathbb{R}}$ are constants (see Fig. 3 for an illustration). The GHM is a local model, which assumes the fibers have no component out of the local tangent plane to the wall (i.e. no imbrication angle). It describes a series of “planes” of fibers along the transmural axis, corresponding to the local heart wall. Thus, the orientation function above allows defining the GHM as a vector field^33^ $v\,:{{\mathbb{R}}}^{3}\to {{\mathbb{R}}}^{3}$:

2 $$v(x,y,z)=(\cos (\theta (x,y,z)),\,\sin (\theta (x,y,z)),0)$$

which is illustrated for several parameter values in Fig. 4 using 3D streamlines.

Recent works using diffusion imaging suggest that |*k*_*N*_| and |*k*_*T*_| are quite small across species, whereas *k*_*B*_ is always distinctly non-zero^4,33^. In the present article, therefore, we also analyze a GHM where the in-plane curvatures vanish (i.e. *k*_*T*_ = *k*_*N*_ = 0).

### Stochastic Calculus on the Cardiac Manifold

Previous work by Young and Panfilov^17^ has modeled diffusion propagation of the electrophysiological signal responsible for cardiomyocyte contraction using Riemannian geometry. The essence of this approach is to consider distance measures in the heart wall to be warped by the local anisotropy of the fibers.

Let $({{\mathbb{R}}}^{3},g)$ be a Riemannian manifold. Consider the following moving frame field to be a basis for the tangent space:

3 $$[\begin{array}{c}{\hat{e}}_{1}\\ {\hat{e}}_{2}\\ {\hat{e}}_{3}\end{array}]=[\begin{array}{ccc}\cos (\theta ) & \sin (\theta ) & 0\\ -\sin (\theta ) & \cos (\theta ) & 0\\ 0 & 0 & 1\end{array}]$$

Then, in this dynamic frame, the metric tensor is given by:

4 $$\tilde{g}={\rm{diag}}([{v}_{f}^{-2},{v}_{t}^{-2},{v}_{t}^{-2}])$$

which is the inverse of the diffusivity tensor $\tilde{D}$ in local coordinates. In keeping our notation consistent with prior work^17^, we denote the diffusivity entries along the local fiber direction and in the plane orthogonal to the local fiber as ${v}_{f}^{2}$ and ${v}_{t}^{2}$, since they are proportional to the squares of the conduction velocities (e.g.^34^). We further note that previous work utilized an orthotropic metric tensor, with a third diffusion speed constant accounting for the propagation rate within the laminar sheets of the heart^17^. Here, we have simplified our analysis by considering only one speed in directions transverse to the fiber. Empirical measurements^35^ showing that the difference between *v*_*f*_ and transverse propagation speeds were much larger than the difference in speeds within the transverse plane suggest that this simplification is reasonable.

Intuitively, the metric tensor describes infinitesimal lengths and distances on a manifold. In this case, *g* describes the curved space in which the signal moves, where the warping is caused by the presence of the fibers. In other words, instead of explicitly modeling the fibers, we model space itself as being intrinsically curved, such that the same movement in different directions leads to different distances being covered (with movement faster along fibers than orthogonal to them).

On the cardiac manifold, it is possible to describe diffusion as a continuous space-time stochastic process using the Ito calculus, which generalizes classical calculus to handle random variables. This is in contrast to the standard method of analyzing diffusion in the heart via partial differential equations (PDEs), as it allows probabilistic interpretations of propagation behavior that deterministic approaches do not. Informally, the PDEs approach describes the macroscopic behavior of diffusion, while the stochastic representation describes the nature of a single infinitesimal member of the ensemble that combines to generate the macroscopic average behavior. Another advantage is that the stochastic description of diffusion can permit intuitive and analytic understanding, whereas the PDEs model is generally analytically intractable.

First, the Laplace-Beltrami operator on a Riemannian manifold (the analogue of the Laplacian in Euclidean space) can be defined in local coordinates by

5 $${{\rm{\Delta }}}_{g}f=\frac{1}{\sqrt{|{\rm{\det }}\,g|}}\frac{\partial }{\partial {x}^{j}}(\sqrt{|{\rm{\det }}\,g|}\,{g}^{ij}\frac{\partial f}{\partial {x}^{i}})$$

where *g*^*ij*^ are elements of the inverse metric tensor *g*^−1^. Note that the Einstein summation convention is used to sum over repeated indices. Then, diffusion on a Riemannian manifold is a stochastic process with an infinitesimal generator given by ${\mathscr{A}}={{\rm{\Delta }}}_{g}/2$^36,37^. Further, define the drift coefficient ${b}^{i}={g}^{jk}{{\rm{\Gamma }}}_{jk}^{i}$ and diffusion coefficient ${\rm{\sigma }}=\sqrt{{g}^{-1}}$, where ${{\rm{\Gamma }}}_{jk}^{i}$ are the Christoffel symbols. Then the following system of stochastic differential equations (SDEs) describes a diffusion process on the manifold^37,38^:

6 $$d{X}_{t}^{i}={{\rm{\sigma }}}_{j}^{i}({X}_{t})\,d{B}_{t}^{j}-\frac{1}{2}{b}^{i}({X}_{t})\,dt$$

where *i* ∈ {1, 2, 3} and *B*_*t*_ is a three-dimensional Wiener process, i.e. ${B}_{t}^{i}\sim {\mathscr{N}}\mathrm{(0},t)$. The Ito process defined by this system of SDEs describes the behavior of diffusion on the cardiac manifold.

### Diffusion on the Cardiac Manifold

Classically, the propagation of the contraction signal in the heart can be modeled as a reaction-diffusion equation, with a spatially varying diffusion tensor *D* (with components *D*^*ij*^) depending on the local fiber orientation^27,28^, via

7 $$\frac{\partial u}{\partial t}=\frac{\partial }{\partial {x}_{i}}{D}_{ij}\frac{\partial u}{\partial {x}_{j}}+{\rm{\Phi }}(u,\overrightarrow{v});\,\frac{\partial \overrightarrow{v}}{\partial t}=\overrightarrow{{\rm{\Psi }}}(u,\overrightarrow{v})$$

where $\overrightarrow{{\rm{\Psi }}}$, Φ are non-linear reaction functions describing the cardiomyocyte electrophysiology, while *u* and $\overrightarrow{v}$ track cellular activation state variables, and the first term is generated by the standard anisotropic diffusion generator $\tilde{ {\mathcal L} }=\frac{\partial }{\partial {x}_{i}}({D}^{ij}\frac{\partial }{\partial {x}_{j}})$. Such studies tend to convert between the diffusion PDE and Riemannian geometric representations by defining the metric to be the inverse diffusivity tensor, with components *D*_*ij*_. However, from a stochastic perspective, it can be shown that a more natural approach is to consider the metric tensor to be the adjugate matrix of the diffusion tensor^39,40^, as it expresses isotropic diffusion in the curved space as anisotropic diffusion in Euclidean space.

**Figure 5:**
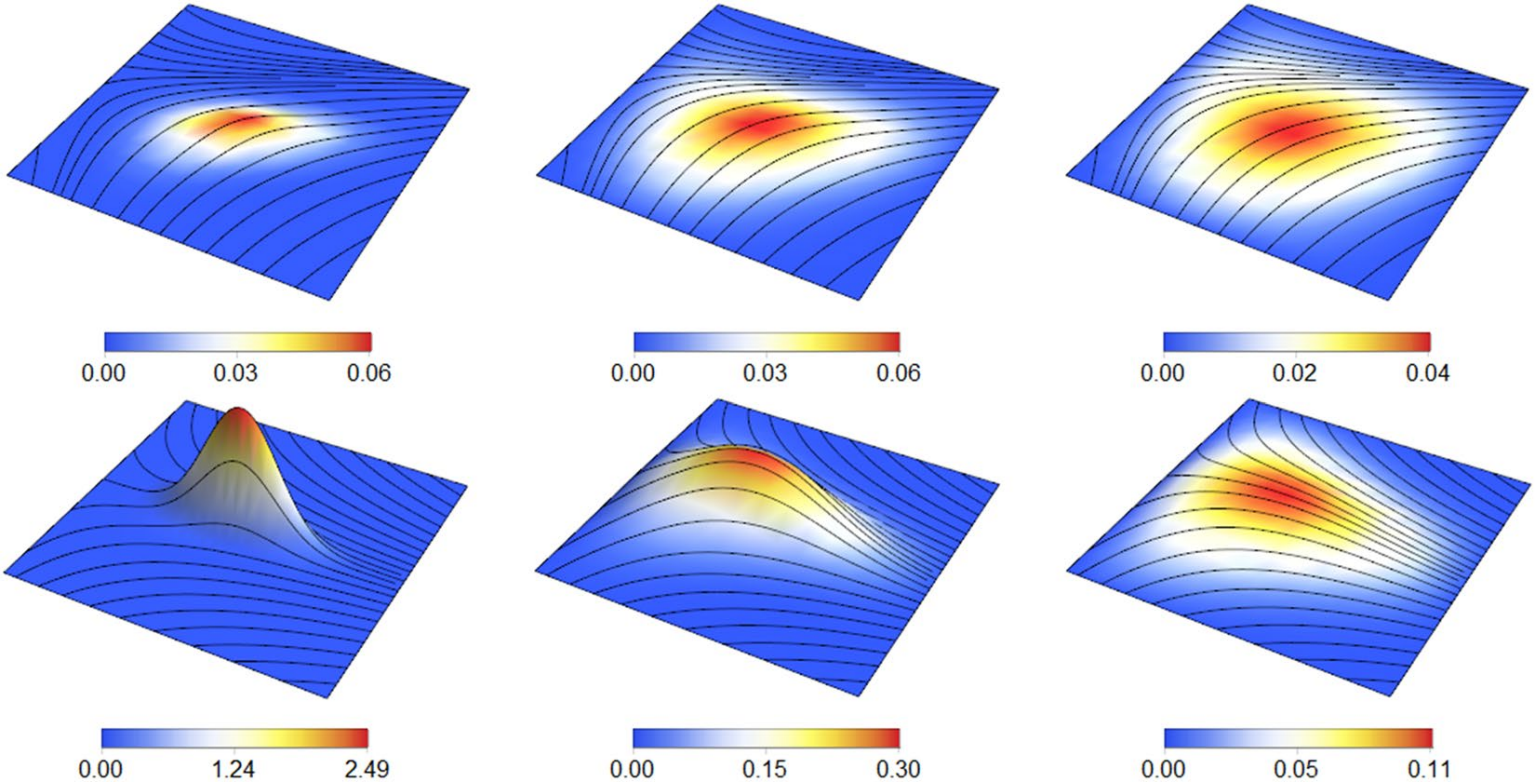
A visualization of the diffusion equation $\frac{\partial u}{\partial t}={{\rm{\Delta }}}_{g}u/2$ on the GHM manifold, with parameters *k*_*N*_ = 0.2, *k*_*B*_ = 0.9, *k*_*T*_ = 0, *v*_*f*_ = 3, *v*_*t*_ = 1. In this example, values of *u* are shown (both in color and in vertical height) for an excitation given by a delta function at (0, 0, 0), approximated by a Gaussian with σ = 0.01, placed at a site on the endocardium (bottom row, *z* = 0) for *t* = 0.1, 0.4, 0.8 (left to right), and for a nearby location just above it in the heart wall (top row, *z* = 1) for *t* = 0.2, 0.6, 1.0. Black lines are the local streamlines of the GHM fibers at that z-slice. The vertical height axis is from 0 to 2.5 for the bottom left 3D inset and from 0 to 0.5 for the rest. The horizontal domain in all cases are *x*, *y* ∈ [−4, 4] × [−4, 4]. Notice the elongating effect of the fibers on the behavior of the propagating signal, as well as the asymmetric spreading due to the fiber fanning due to non-zero *k*_*N*_ (e.g. in the lower-right inset).

There is a deep duality between the heat (isotropic diffusion) equation and BM on the cardiac manifold: the heat kernel, i.e. the fundamental solution to the heat equation $(\frac{\partial }{\partial t}-{\mathscr{A}})u=0$, is exactly the transition density function of BM on the manifold^38,41^. More intuitively, we can formalize the notion that the heat equation describes the expected behavior of an ensemble of BM processes by noting that $u(t,x)={\mathbb{E}}[f({X}_{t,x})]$ solves the manifold heat equation with initial conditions *u*(0, *x*) = *f*(*x*), where *X*_*t*,*x*_ is a BM on the manifold starting from *x*^37,42^. Thus, analysis of the Ito diffusion process above corresponds to understanding the heat equation on the manifold, which comprises the diffusion term in the reaction-diffusion equation above. Figure 5 illustrates how the curved GHM space affects diffusion from a point in a helicoidal medium, such as from an excitation site of the Purkinje network in the endocardium^2^.

Herein, we focus on the behavior of diffusion on the manifold from a stochastic perspective, to understand how the GHM affects contraction signal propagation.

## Results

### The GHM Manifold Possesses Negative Ricci Curvature

As noted above, the cardiac tissue can be considered a Riemannian manifold with a metric tensor defining spatial distances on the manifold *g*, warped by the presence of the fibers. For any such manifold, the curvature of the space can be measured via the Ricci curvature scalar *R*, which in the case of the GHM is given by (*see* *Supplemental Information (Derivations): Ricci Curvature of the Cardiac Manifold*)

8 $$R=\frac{-{k}_{B}^{2}{({v}_{f}^{2}-{v}_{t}^{2})}^{2}}{2{v}_{f}^{2}},$$

in agreement with previous calculations by Young and Panfilov^17^. Since the scalar curvature is spatially constant and always negative, it accelerates diffusion propagation on the manifold.

As also noted in previous work^17,43^, this acceleration can be seen in the fact that, for small *t*, the volume of activated tissue grows as

9 $${\rm{Vol}}({B}_{t})=\frac{4\pi }{3}{v}_{f}{v}_{t}^{2}{t}^{3}(1-\frac{R}{30}{t}^{2}+O({t}^{4}))$$

where *B*_*t*_ is a metric ball at time *t*. We note another connection to the speed of diffusion lies in considering the short-time asymptotic expansion of the heat kernel *p*(*t*, *q*_1_, *q*_2_), the fundamental solution of the diffusion equation $(\frac{\partial }{\partial t}-{\mathscr{A}})u=0$ on the manifold, where $t\in {\mathbb{R}}$ and ${q}_{1},{q}_{2}\in {{\mathbb{R}}}^{3}$. For the GHM manifold, it can be shown that the autodiffusion function *h*_*t*_(*q*) = *p*(*t*, *q*, *q*) can be expanded as^42,44^

10 $${h}_{t}(q)=\frac{1}{{(2\pi t)}^{3/2}}(1+\frac{R}{12}t+O({t}^{2}))$$

when *t* is small, showing that the local Ricci scalar curvature dominates the local behavior of diffusion on such time scales. Recalling that *p* is the transition density of the Ito diffusion defining BM on the manifold, we again see the duality between the stochastic and PDE formulations of the signal diffusing. One can thus interpret *h*_*t*_(*q*) as either the flow of a quantity from a point to itself or the probability of BM staying at *q* over time. Informally, if the probability of staying near a start point *q* (i.e. *h*_*t*_(*q*)) is lower, then the diffusion is flowing away from its start point *q* more quickly. As such, in the case that *R* < 0, notice that the curvature term decreases the value of *h*_*t*_(*q*), meaning that the term is encouraging the diffusion flow to move faster from its start point. Hence, negative *R* relates directly to faster diffusion from a point.

A natural question is whether the GHM optimizes the Ricci curvature in the variational sense, which would imply a maximization or minimization of the curvature-derived acceleration of the diffusion rate. Using the calculus of variations, we treat the Ricci curvature as a functional on the space of orientation functions. Akin to classical calculus, the first variation shows whether an input is an extremal point in function space, while the second variation classifies its type. We consider two different function spaces: the set of “planar” fiber fields (with no imbrication angle) and the space of general 3D unit vector fields.

Using variational techniques, it can be shown that the GHM lies at a stationary point of the Ricci curvature scalar, in the planar case (*see* *Supplemental Information (Derivations): Variational Analysis of the GHM Ricci Curvature*). In contrast, in the general space, only the GHM with *k*_*N*_ = *k*_*T*_ = 0 has vanishing first variation; the full GHM is not a stationary point for other parameter values. However, the stationary point is neither a minimum nor a maximum, as it fails to satisfy the Legendre condition, which is a necessary requirement for the function to lie at an extremum^45^.

We note that the variational status of the GHM as a stationary point with respect to the Ricci curvature in the full space requires the vanishing of the in-plane curvatures (*k*_*N*_ and *k*_*T*_). Recent imaging studies with GHM fits confirm that these curvatures are small in magnitude^4,33^. Another example of the importance of the vanishing of these curvatures will be shown in the next section, with respect to the drift vector of diffusion on the manifold.

In summary, corroborating previous analyses^17^, the GHM has a constant negative Ricci curvature, which has an accelerating effect on the diffusion process. Our variational analysis of the GHM shows that it always lies at a stationary point in the planar space of fiber angle functions, but it is only stationary in the full space when the in-plane curvatures (*k*_*N*_, *k*_*T*_) vanish. It is interesting that the GHM lies only at a stationary point, rather than a minimum or maximum, perhaps indicating a balance between competing factors, such as wave speed and stability. We note that previous studies^22,23,26^ suggest that increased rate of transmural rotation (determined by *k*_*B*_) may be linked to cardio-electrophysiological instability. Hence, although increasing |*k*_*B*_| would accelerate the signal propagation, this may not be beneficial to the organism overall.

### The Diffusion Drift is Minimized by the Helicoidal Architecture

The Ito diffusion process of BM on a manifold is described by a system of SDEs that is parametrized by a diffusion coefficient ${\rm{\sigma }}=\sqrt{{g}^{-1}}$ and drift coefficient ${b}^{i}={g}^{jk}{{\rm{\Gamma }}}_{jk}^{i}$. For the GHM, the former is given by

11 $$\sigma =\frac{1}{2}[\begin{array}{ccc}\varsigma +\delta \,\cos (2\theta ) & \delta \,\sin (2\theta ) & 0\\ \delta \,\sin (2\theta ) & \varsigma -\delta \,\cos (2\theta ) & 0\\ 0 & 0 & 2{v}_{t}\end{array}]$$

where *ς* = *v*_*f*_ + *v*_*t*_ and *δ* = *v*_*f*_ − *v*_*t*_, while the latter can be written as

12 $${b}^{i}=\frac{\delta \varsigma [\,-\,{\alpha }_{i}\,\cos \,\mathrm{(2}\theta )+{\beta }_{i}\,\sin \,\mathrm{(2}\theta )]}{1+2{k}_{N}x-2{k}_{T}y+\gamma ({x}^{2}+{y}^{2})}$$

and *b*^3^ = 0, where $\gamma ={k}_{N}^{2}+{k}_{T}^{2}$, which intuitively measures the total local in-plane fiber curvature, *α*_1_ = −*β*_2_ = *k*_*N*_ + *γx*, and *α*_2_ = *β*_1_ = *k*_*T*_ − *γy*. Thus, to determine when the drift components vanish across the manifold, consider the Euclidean magnitude (i.e. sum of squared components) of the drift vector for the GHM:

13 $$\parallel b{\parallel }_{2}^{2}=\frac{\gamma {({v}_{f}^{2}-{v}_{t}^{2})}^{2}}{1+2{k}_{N}x-2{k}_{T}y+\gamma ({x}^{2}+{y}^{2})}$$

so that $\parallel b{\parallel }_{2}^{2}=0$ clearly implies ${k}_{N}^{2}+{k}_{T}^{2}=0$, which holds only when *k*_*N*_ = *k*_*T*_ = 0. As such, mathematically, the drift vector is zero across the manifold if and only if *k*_*N*_ = *k*_*T*_ = 0, assuming *v*_*f*_ > *v*_*t*_. Hence, the empirical observation^4,33^ that *k*_*N*_ and *k*_*T*_ are small in nature (essentially equivalent to the assumptions of the rule-based model^32^) can be interpreted as a vanishing of the drift vector (i.e. deterministic bias) in the Ito process.

The calculus of variations can also provide insight into the minimization of the magnitude of the drift vector. It can be shown (*see* *Supplemental Information (Derivations): Variational Analysis of the Ito Diffusion Drift on the GHM Manifold*) that, in its most general form, the GHM does not satisfy the Euler-Lagrange equations for the Riemannian norm of the drift vector. Yet, if the in-plane curvatures vanish (i.e. *k*_*N*_ = *k*_*T*_ = 0), the GHM does lie at a minimum, since the drift is identically zero. Hence, in the space of fiber angle functions, the minimization of the diffusion drift is linked to the vanishing of the in-plane curvatures, similar to the case of the Ricci curvature.

Thus, we have shown two results linking the in-plane curvatures of the GHM to the drift vector. The first shows that the drift vector is identically zero across the cardiac manifold generated by the GHM if and only if the in-plane curvatures vanish. The second is a complementary result, showing that the full GHM (i.e. with arbitrary in-plane curvatures) is not generally a variational minimizer of the drift magnitude, unless *k*_*N*_ and *k*_*T*_ vanish. As such, the empirical findings^4,33^ that the curvature parameters *k*_*T*_, *k*_*N*_ in the tangential plane to the mammalian heart wall are small relates also to the minimization of the Ito drift.

### In-Plane Variance Becomes Isotropic Exponentially Quickly

The results above suggest that several special properties of the GHM rely on the vanishing of the in-plane curvatures, *k*_*T*_ and *k*_*N*_. In this section, we restrict to this case, and focus on the interpretation of the transmural rotation parameter *k*_*B*_.

Although there is no deterministic drift (or convection) component to the GHM encountered empirically (i.e. that with vanishing in-plane curvatures, or *k*_*N*_ = *k*_*T*_ = 0), there is still a stochastic bias in the components of the variance vector so that diffusion in some directions is favored over others. We show that for the GHM, as long as *k*_*B*_ ≠ 0, in the heart wall tangent (*xy*) plane, this bias is removed exponentially quickly. We also show that the case of *k*_*B*_ = 0, corresponding to no transmural rotation, is biologically problematic, because then the variance vector has a strong constant bias in the fiber direction *x*.

For the GHM with *k*_*N*_ = *k*_*T*_ = 0, we obtain the following system of SDEs:

14 $$\begin{array}{rcl}d{X}_{s}^{1} & = & \frac{1}{2}([\varsigma +\delta \,\cos (2{k}_{B}{X}_{s}^{3})]\,d{B}_{s}^{1}+\delta \,\sin (2{k}_{B}{X}_{s}^{3})\,d{B}_{s}^{2})\\ d{X}_{s}^{2} & = & \frac{1}{2}(\delta \,\sin (2{k}_{B}{X}_{s}^{3})\,d{B}_{s}^{1}+[\varsigma -\delta \,\cos (2{k}_{B}{X}_{s}^{3})]\,d{B}_{s}^{2})\\ d{X}_{s}^{3} & = & {v}_{t}\,d{B}_{s}^{3}\end{array}$$

See Fig. 2 (left) for sample trajectories obtained by numerically integrating the SDE above. One can see the influence of fibers on the diffusion processes, analogous to the acceleration along fibers seen in Fig. 1 (right).

**Figure 6:**
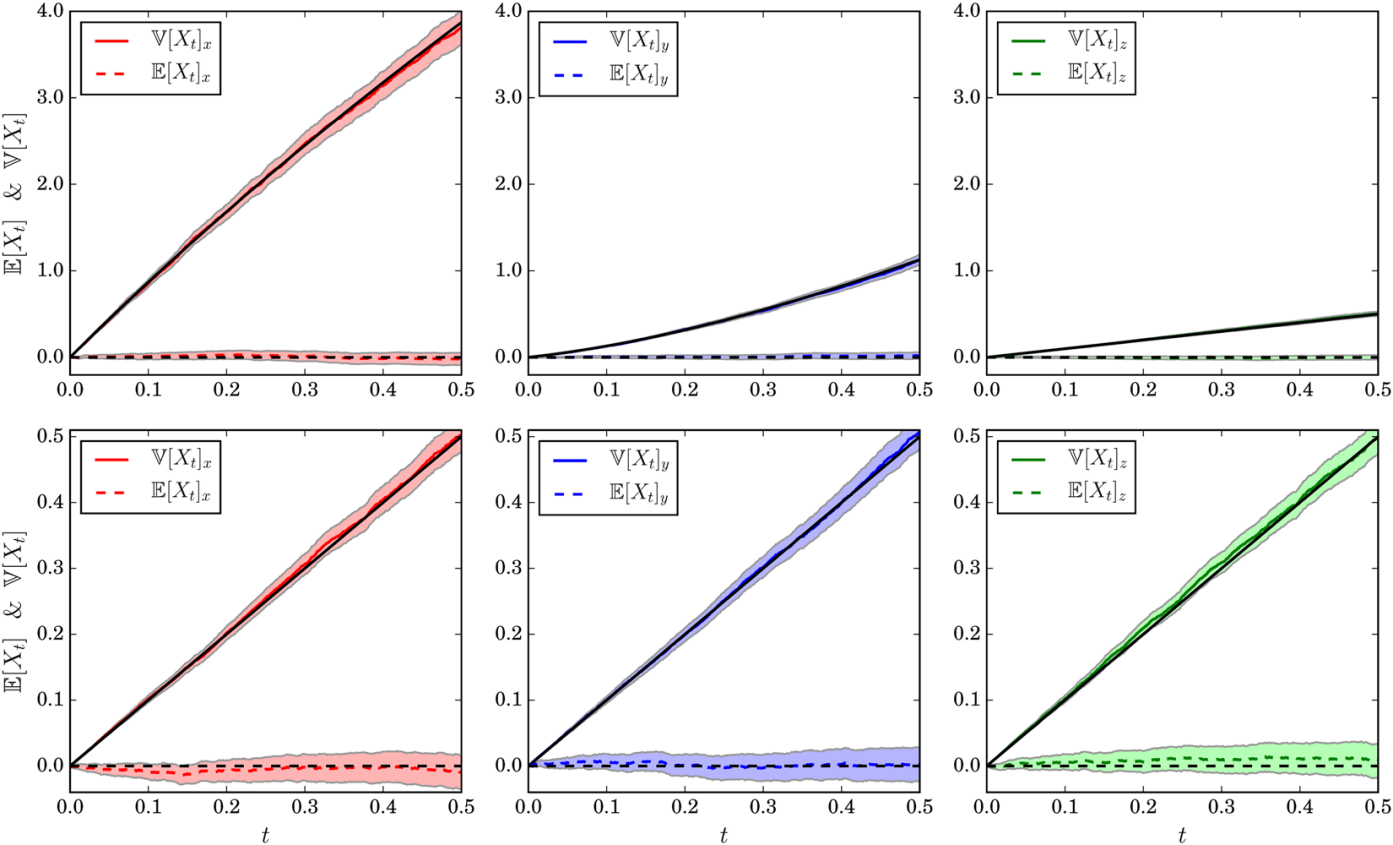
Variance (solid lines) and means (dashed lines) over time for the *x* (red/left), *y* (blue/middle), and *z* (green/right) directions, respectively. Black lines are theoretical predictions. Top row: *v*_*f*_ = 3, *v*_*t*_ = 1; bottom row: *v*_*f*_ = 1, *v*_*t*_ = 1 (i.e. isotropy). Shaded regions are 99% confidence intervals using large-sample normal approximations to the sampling distributions of the sample mean and variance. Simulation parameters are the same as in Fig. 1 (bottom right), run 5000 times.

It can be shown (*see* *Supplemental Information (Derivations): Moments of the GHM Stochastic Diffusion Process*) that the variance of the stochastic process described by these SDEs is given by:

15 $${\mathbb{V}}[{X}_{t}]=t\,[\begin{array}{c}\raisebox{1ex}{$({v}_{f}^{2}+{v}_{t}^{2})$}\!\left/ \!\raisebox{-1ex}{$2$}\right.\\ \raisebox{1ex}{$({v}_{f}^{2}+{v}_{t}^{2})$}\!\left/ \!\raisebox{-1ex}{$2$}\right.\\ {v}_{t}^{2}\end{array}]+\frac{\delta \varsigma [1-\exp (\,-\,ct)]}{4{k}_{B}^{2}{v}_{t}^{2}}[\begin{array}{c}1\\ -1\\ 0\end{array}]$$

where $c=2{k}_{B}^{2}{v}_{t}^{2}$ and that the expected value is /constant at zero. See Fig. 6 for plots of the numerical convergence of the moments of the process. Notice that the first term of the variance vector has identical components in the two in-plane directions *x*, *y* in the heart wall tangent plane, with a smaller component in the transmural direction *z*. The second term has components with identical magnitudes in the fiber direction *x* and the in-plane direction *y*, but with the latter having a minus sign. This causes an increase in variance in the *x* direction with a corresponding decrease in the *y* direction. Since the first term in the variance equation above has no dependence on *k*_*B*_, it is in fact the second term that relates to the directional bias to the variance vector caused by the transmural rotation of fibers.

Now, recall that the variance of a diffusion process can be interpreted as being related to the expected growth (movement) of the diffusion in that direction. For instance, the variance of BM in 1D isotropic Euclidean space (${\mathbb{R}}$) is ${\mathbb{E}}[{({B}_{t})}^{2}]=t$; i.e. linear in time. Hence, we can gain intuition about the behavior of diffusion on the GHM by examining the asymptotics of the variance as we vary some of the parameters of the GHM diffusion model. As a simple case, suppose that *v*_*f*_ = *v*_*t*_. Then, as one would expect, ${\mathbb{V}}[{X}_{t}]=t{v}_{t}^{2}{\mathrm{[1},1,\mathrm{1]}}^{T}$, meaning the directional anisotropy in the variance vector disappears. In particular, if *v*_*f*_ = *v*_*t*_ = 1, we get the classical result for Brownian diffusion in ${{\mathbb{R}}}^{3}$. Next, consider the case where *k*_*B*_ → 0, so that there is no transmural rotation of fibers in the heart wall. It can be shown (*see* *Supplemental Information (Derivations): Moments of the GHM Stochastic Diffusion Process*) that ${\mathrm{lim}}_{{k}_{B}\to 0}\,{\mathbb{V}}[{X}_{t}]=t{[{v}_{f}^{2},{v}_{t}^{2},{v}_{t}^{2}]}^{T}$, meaning that growth in the variance vector is accelerated in the fiber direction *x*, whereas it is not in *y* and *z*.

We next consider the asymptotic behavior of the terms of the variance vector in time. As *t* increases, the first variance term grows linearly, while the second converges exponentially fast to a constant. This can be interpreted as an isotropic linear growth of the variance vector in the heart wall tangent plane (*xy*) in time, with a reshaping to increase the variance of the diffusion process along fibers in the direction *x* while decreasing it across them in the direction *y*. However, the effect of this reshaping becomes quickly negligible as time increases. We also observe that this effect is identical to that of increasing *k*_*B*_, the helicoidal fiber rotation rate. Intuitively, a larger *k*_*B*_ means greater homogeneity in *x* and *y*, converging to a growth averaging the diffusion rates in the fiber and non-fiber directions. Thus:

16 $$\mathop{{\rm{l}}{\rm{i}}{\rm{m}}}\limits_{t\to {\rm{\infty }}}\,{\mathbb{V}}[{X}_{t}]\approx t\,[\begin{array}{c}\raisebox{1ex}{$({v}_{f}^{2}+{v}_{t}^{2})$}\!\left/ \!\raisebox{-1ex}{$2$}\right.\\ \raisebox{1ex}{$({v}_{f}^{2}+{v}_{t}^{2})$}\!\left/ \!\raisebox{-1ex}{$2$}\right.\\ {v}_{t}^{2}\end{array}]=\mathop{{\rm{l}}{\rm{i}}{\rm{m}}}\limits_{{k}_{B}\to {\rm{\infty }}}\,{\mathbb{V}}[{X}_{t}]$$

as long as *k*_*B*_, *v*_*t*_, *v*_*f*_ ≠ 0.

Hence, in the heart wall tangent plane, the diffusion variance asymptotically expands at the average of the accelerated and non-accelerated squared rates, $({v}_{f}^{2}+{v}_{t}^{2})/2$; i.e. growth expands at the same rate in the *x* and *y* directions. Intuitively, the fibers create a local directional bias initially, but this vanishes exponentially fast into an isotropically accelerated propagation pattern in the heart wall tangent plane. We also showed that the bias of the variance vector grows linearly in time when *k*_*B*_ → 0, which is the case where all fibers lie along the *x*-axis (i.e. no transmural turning). There is strong empirical evidence that *k*_*B*_ is non-zero (i.e. that there is always transmural turning of fibers)^4,31–33^. Thus, similar to how the empirical minimization of *k*_*T*_ and *k*_*N*_ can be interpreted as minimization of the drift vector *b*, the presence of non-zero *k*_*B*_ can be viewed as reducing the bias of the variance vector within the heart wall plane.

Our expression for the variance function allows us characterize the timescale of the exponential convergence. We can consider the difference between the *x* and *y* components of the variance as the in-plane stochastic bias: ${\mathbb{V}}{[{X}_{t}]}_{x}-{\mathbb{V}}{[{X}_{t}]}_{y}=\delta \varsigma \mathrm{[1}-\exp (\,-\,ct)]/\mathrm{(2}{k}_{B}^{2}{v}_{t}^{2})$. This difference can be seen to converge to a constant ${c}_{\infty }=\delta \varsigma /\mathrm{(2}{k}_{B}^{2}{v}_{t}^{2})$ in time, with exponential coefficient $c=2{k}_{B}^{2}{v}_{t}^{2}$. Thus, the time constant of decay is given by *τ* = *c*^−1^. To give biophysically plausible values, we use past fits of GHM parameters in the human heart^4^, which show that *k*_*B*_ = −0.17 rad/mm, and measurements of cardiac conduction velocities, which suggest a ratio of diffusivities (i.e. ratio of squared wave velocities along vs across fibers) of approximately 4 in the ventricles^46^. We used a diffusivity along fibers of ${v}_{f}^{2}=3\times {10}^{-4}$ m^2^/s, following a previous model^34^, so that ${v}_{t}^{2}={v}_{f}^{2}/4=7.5\times {10}^{-5}$ m^2^/s. As a caveat, we note that measurement of the conductivities is itself a difficult problem, and there is variability in the values reported in the literature^47,48^ as well as spatially in the heart wall^46^. With these values, we can estimate that *c*_∞_ = 5.19 × 10^−5^ m^2^ and *τ* = 0.23 s. This result suggests that, while the shrinking of the in-plane stochastic bias is exponential, it seems plausible that at least some effect on the wave front due to the anisotropy can persist for a non-trivial fraction of the heartbeat. Uncertainty in the measurement of diffusivities as well as their spatial variation preclude surety in our conclusion. After approximately *τ* = 0.23 seconds have passed, the ratio of the magnitude of the *x* (or *y*) component of the first term in the variance equation to the magnitude of the *x* (or *y*) component of its second term, is 2.63, meaning that the first (isotropic) term dominates.

In summary, based on our results concerning the drift vector, we restricted our attention to the GHM with vanishing in-plane curvatures, and considered the variance of the diffusion process, which can serve as a measure of its growth rate along each dimension. We showed the stochastic bias of this process in the heart wall tangent plane vanished exponentially quickly, as long as *k*_*B*_ ≠ 0. In combination with our results showing the vanishing of the deterministic bias (i.e. Ito drift), the shrinking of the anisotropy of the variance vector in the heart wall tangent plane suggests that the helicoidal fiber geometry found in mammalian hearts^4,33^, where *k*_*N*_, *k*_*T*_ ≈ 0 and *k*_*B*_ ≠ 0, minimizes the diffusion bias.

## Discussion

The contraction of myocytes in the heart wall is controlled by current flowing through an elaborate Purkinje network, which raises the question of how current diffuses through the wall around the insertion points. Classically, the propagation of the contraction signal is represented using a reaction-diffusion equation with the inverse diffusivity tensor as its metric^17,27,28^. We combined this Riemannian approach with a minimal surface model of the cardiac fiber geometry, the generalized helicoid model (GHM)^4^, and exploited the Ito calculus to describe and analyze diffusion on it.

Among our results, we showed that the helicoidal structure of cardiac fibers minimizes local biases in the diffusion, both deterministic (embodied by the Ito drift vector) and stochastic (derived from differing *x* and *y* components of the second moment of the Ito diffusion SDEs). Since the GHM is a local approximation anywhere in the left ventricular wall^4^, one would expect it to not favor biased flow in a particular direction. In line with these theoretical expectations, we find that the GHM with *k*_*N*_ = *k*_*T*_ = 0 minimizes the Ito drift, providing a natural interpretation for the recent imaging studies that suggest |*k*_*N*_| and |*k*_*T*_| are relatively small across species^4,33^. Moreover, using the variance of the Ito diffusion on the cardiac manifold with vanishing in-plane curvatures, we also show that stochastic bias in the heart wall tangent plane shrinks exponentially fast in time, becoming isotropic with a rate given by averaging the squares of the speeds along and across fibers.

This solution is also good in a functional analytic sense, for both the drift and Ricci curvature. Using the calculus of variations, we showed that the GHM lies at a stationary point of the Ricci curvature in the space of orientation functions, provided the in-plane curvatures are zero. However, it is neither a minimum nor a maximum. One might speculate that this is because increased negativity of the Ricci scalar, equivalent to increased transmural rotation rate (or |*k*_*B*_| for the GHM) could lead to cardiomyopathic disturbances in the signal wavefront as it propagates^26^, such as scroll wave turbulence^22,23^. On the other hand, there are a number of potential mechanical reasons for the presence of the helicoidal structure, including shear wave filtering^49,50^, energy dissipation^6^, and mechanical efficiency^11,29^. Thus, the competing effects of changing *k*_*B*_ must be balanced to allow safe and fast wave propagation, as well as mechanical efficacy. Physical constraints, such as the myocyte size and necessary accompanying extracellular matrix structure, which idealized models often do not capture, also affect the structure.

While we have considered a number of properties conferred upon the heart by its helicoidal geometry, there are a few limitations inherent to our approach. Currently, we consider only local, static fiber geometry within the left ventricle. Our analysis also only considers the diffusion term in the reaction-diffusion contraction wave propagation equation, since this is the term that is affected by myocyte orientation and it largely governs the local spreading of the signal. In addition, we do not consider the effect of cardiomyocyte laminar sheets, which may contribute to both the electrophysiology^17,51^ and mechanics^52^ of the heart. Our approach provides useful insights, despite these approximations, because the effect of the fibers on diffusion of the contraction signal has been empirically shown to be much greater than that of the sheets^35^. Lastly, the GHM assumes that fibers lie in planes tangent to the heart wall. Although this is an acceptable local approximation^4,33^, some studies suggest that the presence of fibers with an out-of-plane component may play a role in cardiac function^53^. Finally, exploring the link between local diffusion bias and long-term wave propagation in the heart is a promising avenue for future work.

In the end, our analysis reveals that the GHM arrangement minimizes any in-plane directional biases in the diffusion process, provided that there is transmural rotation and small in-plane curvature of the fibers in the heart wall. Normally, one might have thought about the orientation of fibers from a purely mechanical perspective. However, given the ubiquity of helicoidal arrangements in biology and artificial composites^5–9^, it should perhaps not be a surprise that nature has used this geometry to satisfy both the mechanical and electrical requirements of the heart.

## Electronic supplementary material

Supplemental Information
