# Supplementary material for: Conduction in the Heart Wall: Helicoidal Fibers Minimize Diffusion Bias
Source: Sci Rep. 2018 May 8;8:7165. doi: 10.1038/s41598-018-25334-7 (PMC5940931; doi:10.1038/s41598-018-25334-7)
Supplement: Supplementary file 1 — Supplemental Information [file 41598_2018_25334_MOESM1_ESM.pdf]

Conduction in the Heart Wall: Helicoidal Fibers Minimize Diffusion Bias

Supplemental Information (Derivations)

Tristan Aumentado-Armstrong[1], Amir Kadivar[1,4], Peter Savadjiev[1,2], Steven W. Zucker[3], and Kaleem Siddiqi[1,*]

[1]School of Computer Science and Center for Intelligent Machines, McGill University, Canada
[2]Department of Diagnostic Radiology, McGill University, Canada
[3]Department of Computer Science and Department of Biomedical Engineering, Yale University, USA
[4]Department of Mathematics and Statistics, McGill University, Canada
[*]siddiqi@cim.mcgill.ca

Ricci Curvature of the Cardiac Riemannian Manifold

We consider the GHM with $k_T = k_N = 0$. Hence, $\theta = k_B z$. Denote $\varsigma = v_f + v_t$, $\delta = v_f - v_t$, and $(x_1, x_2, x_3) = (x, y, z)$. We first transform the metric tensor to the static coordinate frame $F = \{\hat{i}, \hat{j}, \hat{k}\}$. Using the following orthogonal matrix:

$$A = \begin{bmatrix} \cos(\theta) & \sin(\theta) & 0 \\ -\sin(\theta) & \cos(\theta) & 0 \\ 0 & 0 & 1 \end{bmatrix}$$

The new metric tensor is given by $g = A^T \widetilde{g} A$:

$$g = \begin{bmatrix} \dfrac{\cos^2(\theta)}{v_f^2} + \dfrac{\sin^2(\theta)}{v_t^2} & g_{12} & 0 \\ g_{21} & \dfrac{\cos^2(\theta)}{v_t^2} + \dfrac{\sin^2(\theta)}{v_f^2} & 0 \\ 0 & 0 & \dfrac{1}{v_t^2} \end{bmatrix}$$

where

$$g_{12} = g_{21} = \frac{\cos(\theta)\sin(\theta)}{v_f^2} - \frac{\sin(\theta)\cos(\theta)}{v_t^2}$$

as well as the inverse metric tensor:

$$g^{-1} = \frac{1}{2} \begin{bmatrix} \omega + \varsigma\delta\cos(2\theta) & \varsigma\delta\sin(2\theta) & 0 \\ \varsigma\delta\sin(2\theta) & \omega - \varsigma\delta\cos(2\theta) & 0 \\ 0 & 0 & 2v_t^2 \end{bmatrix}$$

where $\omega = v_f^2 + v_t^2$. We can compute the Christoffel symbols of the second kind:

$$\Gamma^m_{ij} = g^{mk} \frac{1}{2} \left(\frac{\partial g_{ki}}{\partial x^j} + \frac{\partial g_{kj}}{\partial x^i} - \frac{\partial g_{ij}}{\partial x^k} \right)$$

as well as the Riemann curvature tensor:

$$R^\ell_{ijk} = \frac{\partial}{\partial x^j}\Gamma^\ell_{ik} - \frac{\partial}{\partial x^k}\Gamma^\ell_{ij} + \Gamma^\ell_{js}\Gamma^s_{ik} - \Gamma^\ell_{ks}\Gamma^s_{ij}$$

Finally, using these, we may compute the Ricci curvature tensor $R_{ij} = g^{\ell m} R_{i\ell jm}$, given here by:

$$\begin{aligned}
R_{00} &= \frac{-k_B^2(v_f^4 - v_t^4)[v_t^2 - v_f^2 + (v_f^2 + v_t^2)\cos(2\theta)]}{4v_f^4 v_t^2} \\
R_{11} &= \frac{k_B^2(v_f^4 - v_t^4)[-v_t^2 + v_f^2 + (v_f^2 + v_t^2)\cos(2\theta)]}{4v_f^4 v_t^2} \\
R_{22} &= \frac{-k_B^2(v_f^2 - v_t^2)^2}{2v_f^2 v_t^2} \\
R_{01} = R_{10} &= \frac{-k_B^2(v_f^2 - v_t^2)[v_f^2 + v_t^2]\sin(2\theta)}{4v_f^4 v_t^2} \\
R_{02} = R_{20} &= R_{21} = R_{12} = 0
\end{aligned}$$

Then the Ricci curvature scalar $R = R^i_i = g^{ij} R_{ij}$ can be given by:

$$R = \frac{-k_B^2(v_f^2 - v_t^2)^2}{2v_f^2}$$

This is consistent with the calculations given previously[1]. Note that the Ricci curvature R for the full GHM (i.e. k_T, $k_N \neq 0$) is identical to that of the case with vanishing in-plane curvatures.

Variational Analysis of the GHM Ricci Curvature

We analyze the Ricci curvature of the GHM manifold from a variational perspective, in two function spaces: a planar space, in which the fibers do not have an out-of-plane component, and a full 3D space, where a general smooth unit vector field is considered to represent the fibers.

Planar Space Analysis

We consider whether the GHM is an extremum with respect to the Ricci curvature in the space of in-plane fiber vector functions, using the calculus of variations. First, consider an arbitrary $\theta : \mathbb{R}^3 \to \mathbb{R}$ angle function (e.g. for which the GHM is one specific case), which gives rise to the following arbitrary in-plane fiber geometry:

$$f_1 = (\cos(\theta), \sin(\theta), 0)$$

Then, based on this fiber distribution, the Ricci scalar can be written:

$$R(x,y,z;\theta) = \frac{-\varsigma\delta}{2v_f^2}\left[\varsigma\delta\theta_z^2 + 2v_f^2(\sin(2\theta)\Psi_1 + \cos(2\theta)\Psi_2)\right]$$

where subscripts denote partial differentiation and

$$\Psi_1 = -4\theta_x\theta_y - \theta_{xx} + \theta_{yy}$$
$$\Psi_2 = 2(\theta_y^2 - \theta_x^2 + \theta_{xy})$$

Our variational functional, parametrized by the function θ, is then given by:

$$J[\theta] = \iiint\limits_{\Omega} R(x,y,z;\theta,\partial_i\theta,\partial_i\partial_j\theta)\,dV$$

where $\partial_i\theta$ and $\partial_i\partial_j\theta$ denote the sets of first and second partial derivatives of θ. Thus the Euler-Lagrange equation (Theorem 1 below) is:

$$0 = \frac{\delta J}{\delta\theta} = \frac{\partial R}{\partial\theta} - \sum_{i=1}^{3}\frac{\partial}{\partial x_i}\frac{\partial R}{\partial\theta_i} + \sum_{j=1}^{3}\sum_{k=j}^{3}\frac{\partial^2}{\partial x_j\partial x_k}\left(\frac{\partial R}{\partial\theta_{jk}}\right) = \frac{(v_f^2 - v_t^2)^2}{v_f^2}\theta_{zz}$$

which clearly holds when θ represents the GHM. By Theorem 1, this means that if we constrain the value of θ on the boundary of Ω, the GHM exists at a local stationary point in the function space.

An obvious question is whether this stationary point is a maximum, minimum, or neither, with respect to the functional J. To answer this question, we use the Legendre condition, which is a necessary requirement for a given function to lie at an extremum of the functional. Since our functional J relies on the second derivatives of the input function θ, the Legendre condition does as well. However, since R is linear in $\theta_{xx}, \theta_{xy}, \theta_{yy}$ and not dependent on the other second derivatives. By applying Theorem 2 below, the conditions of which are satisfied here, we obtain the following condition: *a necessary condition for the GHM θ to be a weak local minimum (maximum) of J is that $\mathscr{D}_2[R]$ is positive (negative) semidefinite for all x,y,z where*

$$\mathscr{D}_2[R] = \begin{bmatrix} \frac{\partial^2 R}{\partial\theta_x^2} & \frac{\partial^2 R}{\partial\theta_x\partial\theta_y} & \frac{\partial^2 R}{\partial\theta_x\partial\theta_z} \\ \frac{\partial^2 R}{\partial\theta_y\partial\theta_x} & \frac{\partial^2 R}{\partial\theta_y^2} & \frac{\partial^2 R}{\partial\theta_y\partial\theta_z} \\ \frac{\partial^2 R}{\partial\theta_z\partial\theta_x} & \frac{\partial^2 R}{\partial\theta_z\partial\theta_y} & \frac{\partial^2 R}{\partial\theta_z^2} \end{bmatrix}$$

For the case of the GHM, this condition fails, implying we are neither at a minimum nor a maximum. First, for the second

derivatives with respect to the first partials, we get the following:

$$\frac{\partial^2 R}{\partial \theta_x^2} = 4\varsigma\delta\cos(2\theta) = -\frac{\partial^2 R}{\partial \theta_y^2}$$
$$\frac{\partial^2 R}{\partial \theta_z^2} = -\frac{\varsigma^2\delta^2}{v_f^2}$$
$$\frac{\partial^2 R}{\partial \theta_x \partial \theta_y} = 4\varsigma\delta\sin(2\theta)$$
$$\frac{\partial^2 R}{\partial \theta_x \partial \theta_z} = \frac{\partial^2 R}{\partial \theta_y \partial \theta_z} = 0$$

This implies that the matrix is given by:

$$\mathscr{D}_2[R] = 4\varsigma\delta \begin{bmatrix} \cos(2\theta) & \sin(2\theta) & 0 \\ \sin(2\theta) & -\cos(2\theta) & 0 \\ 0 & 0 & \frac{-\varsigma\delta}{4v_f^2} \end{bmatrix}$$

The eigenvalues of $\mathscr{D}_2[R]$ are then:

$$\text{Eigenvalues}(\mathscr{D}_2[R]) = \frac{-\varsigma^2\delta^2}{v_f^2}, 4\varsigma\delta, -4\varsigma\delta$$

Note that the presence of both negative and positive eigenvalues implies $\mathscr{D}_2[R]$ is neither positive nor negative semi-definite, and hence R can neither be a minimum nor a maximum of the functional J. (We note in passing the interesting fact that this result holds for any in-plane fiber geometry, not just the GHM).

Full Space Analysis

Consider the general unit vector fiber field

$$\widetilde{f_1} = (\sin(\phi)\cos(\theta), \sin(\phi)\sin(\theta), \cos(\phi))$$

which may complete into an orthonormal frame field via

$$\widetilde{f_2} = (\cos(\theta)\cos(\phi), \sin(\theta)\cos(\phi), -\sin(\phi))$$
$$\widetilde{f_3} = (-\sin(\theta), \cos(\theta), 0)$$

which forms a moving frame $\widetilde{F} = \{\widetilde{f_1}, \widetilde{f_2}, \widetilde{f_3}\}$. Then our metric tensor is given by $g = A^T \widetilde{g} A$, with $A^T = [\widetilde{f_1}, \widetilde{f_2}, \widetilde{f_3}]$, considering $\widetilde{f_i}$ as column vectors. We may then compute the Christoffel symbols and the Ricci scalar curvature R via g. Then, our variational functional, now parametrized by both ϕ and θ, is given by:

$$J[\theta,\phi] = \iiint\limits_{\Omega} R(x,y,z;\theta,\phi,\partial_i\theta,\partial_i\phi,\partial_i\partial_j\theta,\partial_i\partial_j\phi)\,dV$$

Then the system of Euler-Lagrange equations is

$$0 = \frac{\delta J}{\delta\theta} = \frac{\partial R}{\partial\theta} - \sum_{i=1}^{3}\frac{\partial}{\partial x_i}\frac{\partial R}{\partial\theta_i} + \sum_{j=1}^{3}\sum_{k=j}^{3}\frac{\partial^2}{\partial x_j \partial x_k}\left(\frac{\partial R}{\partial\theta_{jk}}\right)$$
$$0 = \frac{\delta J}{\delta\phi} = \frac{\partial R}{\partial\phi} - \sum_{i=1}^{3}\frac{\partial}{\partial x_i}\frac{\partial R}{\partial\phi_i} + \sum_{j=1}^{3}\sum_{k=j}^{3}\frac{\partial^2}{\partial x_j \partial x_k}\left(\frac{\partial R}{\partial\phi_{jk}}\right)$$

Recall that the full GHM can be represented in terms of this new vector field via

$$(\theta,\phi) = \left(\arctan\left(\frac{k_T x + k_N y}{1 + k_N x - k_T y}\right) + k_B z, \frac{\pi}{2}\right)$$

into which plugging the full GHM into the Euler-Lagrange equations then gives

$$\frac{\delta J}{\delta \theta} = 0$$
$$\frac{\delta J}{\delta \phi} = \frac{-2k_B \varsigma^2 \delta^2}{v_f^2} \frac{[k_T - \gamma y]\cos(\theta) + [k_N + \gamma x]\sin(\theta)}{1 + 2k_N x - 2k_T y + \gamma(x^2 + y^2)}$$

which shows that the full GHM is not a critical (stationary) point of the Ricci curvature functional. Figure 1 shows the landscape of the magnitude of this variation with respect to ϕ over a small spatial locality, as k_N and k_T vary. The map depicts the minimality of this functional derivative's magnitude when the in-plane curvatures k_T, k_N are vanishing, as is the case for the ranges of these parameters found in empirical fits to mammalian heart wall fibers[2].

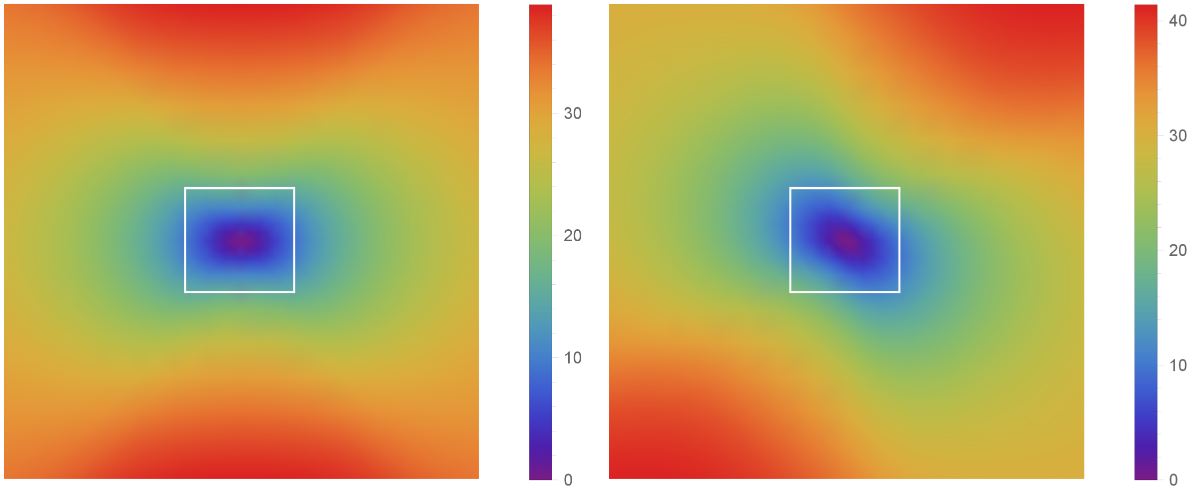

Figure 1. Color plots of $\int\int\int \log\left[1 + s^2\right] dV$, where $s = \frac{\delta J}{\delta \phi}$, integrated over a cube of side-length 2 at the origin (left panel) and at $(0, 0, 0.5)$ (right panel). Values are over $k_T \in [-1, 1]$ on the x-axis and $k_N \in [-1, 1]$ on the y-axis. We fix $k_B = 1$, $v_f = 3$, and $v_t = 1$. The white boxes show the corresponding range of the k_T, k_N values found in empirical fits of the GHM to rat heart fiber data[2], where there is also a very strong concentration at [0,0].

Variational Analysis of the Ito Diffusion Drift on the GHM Manifold

As before, consider the general unit vector fiber field

$$\widetilde{f_1} = (\sin(\phi)\cos(\theta), \sin(\phi)\sin(\theta), \cos(\phi))$$
$$\widetilde{f_2} = (\cos(\theta)\cos(\phi), \sin(\theta)\cos(\phi), -\sin(\phi))$$
$$\widetilde{f_3} = (-\sin(\theta), \cos(\theta), 0)$$

which produces a moving frame $\widetilde{F} = \{\widetilde{f_1}, \widetilde{f_2}, \widetilde{f_3}\}$. Then our metric tensor is given by $g = A^T \widetilde{g} A$, with $A^T = [\widetilde{f_1}, \widetilde{f_2}, \widetilde{f_3}]$, considering $\widetilde{f_i}$ as column vectors. The drift vector is then computed via components defined by $b^i = g^{jk}\Gamma^i_{jk}$, with magnitude computed by $\mathscr{M}(x, y, z; \theta) = ||b||^2 = g_{ij} b^i b^j$. Therefore, we can consider the variational functional given by:

$$J[\theta] = \iiint_\Omega \mathscr{M}(x, y, z; \theta)\, dV$$

Note that, in the case of a planar fiber field (i.e. $\phi \equiv \pi/2$), the drift and its magnitude can be written as

$$b(x, y, z; \theta) = -(v_f^2 - v_t^2) \begin{bmatrix} \cos(2\theta)\theta_y - \sin(2\theta)\theta_x \\ \sin(2\theta)\theta_y + \cos(2\theta)\theta_x \\ 0 \end{bmatrix}$$
$$\mathscr{M}(x, y, z; \theta) = \frac{(v_f^2 - v_t^2)^3}{2 v_f^2 v_t^2} \left[(\eta - \cos(2\theta))\theta_y^2 + (\eta + \cos(2\theta))\theta_x^2 + 2\sin(2\theta)\theta_x\theta_y\right]$$

where $\eta = (v_f^2 + v_t^2)/(v_f^2 - v_t^2)$. We can next consider the Euler-Lagrange equation of the drift magnitude with respect to the fiber field parameters, evaluated at the GHM:

$$0 = \frac{\delta J}{\delta \theta} = \frac{(v_f^2 - v_t^2)^3}{v_f^2 v_t^2} \frac{(k_N - k_T)(k_N + k_T)\sin(2k_B z) + 2k_N k_T \cos(2k_B z)}{(k_N^2 + k_T^2)(x^2 + y^2) + 2k_N x - 2k_T y + 1}$$

$$0 = \frac{\delta J}{\delta \phi} = 2k_B \left(\frac{(v_f^2 - v_t^2)^2 (v_f^2 + v_t^2)}{v_f^2 v_t^2} \right) \left[\frac{(y(k_T^2 + k_N^2) - k_T)\cos(\theta) - (x(k_T^2 + k_N^2) + k_N)\sin(\theta)}{(k_T^2 + k_N^2)(x^2 + y^2) + 2k_N x - 2k_T y + 1} \right]$$

Notice that, assuming $v_f > v_t > 0$ and $k_B \neq 0$, then the above do not hold across the manifold for k_T, k_N non-zero. In particular, consider the Euler-Lagrange equation with respect to ϕ. Assuming it holds at $(x,y,z) = (0,0,0)$, we get that $\theta = 0$ and thus $k_T = 0$. Similarly, at the point $(x,y,z) = (0,0,\pi k_B^{-1}/2)$, vanishing $\delta J/\delta\phi$ implies $k_N = 0$. Conversely, if $k_N = k_T = 0$, we immediately get $\delta J/\delta \phi = 0$.

Since the values of θ are in general not constrained on the boundary of Ω, we also need to check the transversality condition (Theorem 1) to confirm that GHM with $k_N = k_T = 0$ is a stationary point of the functional. Since the choice of the region Ω is arbitrary, the transversality condition is $\left(\frac{\partial}{\partial\theta_x}, \frac{\partial}{\partial\theta_y}, \frac{\partial}{\partial\theta_z}\right)\mathscr{M} = (0,0,0)$ or:

$$(\eta + \cos(2\theta))\theta_x + \sin(2\theta)\theta_y = (\eta - \cos(2\theta))\theta_y + \sin(2\theta)\theta_x = 0$$

which clearly holds for GHM when $k_N = k_T = 0$ in which case we have $\theta_x = \theta_y = 0$.

Hence, for arbitrary k_N and k_T the full GHM is not a variational minimizer of the drift magnitude. However, for the empirically observed parameter values of the heart fiber structure[2] (i.e. $k_N, k_T \approx 0$), the drift magnitude is identically zero, which minimizes the drift.

Moments of the GHM Stochastic Diffusion Process

Using the Ito calculus, we can compute a number of fundamental properties concerning the behavior of the diffusion process. In particular, using the moments of the process, we can provide a description of its asymptotic properties.

First, there are some facts and lemmas we will require. Let $\{B^i\}$ be standard independent Brownian motions. Then:

$$\mathbb{E}[B_t^i] = 0;\ \mathbb{E}[(B_t^i)^2] = t;\ (B_{t+k}^i - B_t^i) \sim \mathscr{N}(0,k) \tag{1}$$

Now suppose $\theta \sim \mathscr{N}(0,\sigma^2)$ is a random variable for some $\sigma \in \mathbb{R}_{>0}$. Then:

$$\mathbb{E}[\sin(\theta)] = 0; \qquad \mathbb{E}[\cos(\theta)] = \exp(-\sigma^2/2) \tag{2}$$

Further, using $\cos^2(\theta) = (1+\cos(2\theta))/2$, we get:

$$\mathbb{E}[\cos^2(\theta)] = (1/2 + \exp(-2\sigma^2)/2) \tag{3}$$

Next, recall the Ito isometry:

$$\mathbb{E}\left[\left(\int_0^T X_t\, dB_t\right)^2\right] = \mathbb{E}\left[\int_0^T X_t^2 dt\right] \tag{4}$$

and the following corollary with two functions using Fubini's theorem:

$$\mathbb{E}\left[\left(\int_0^T f(s)\, dB_s\right)\left(\int_0^T g(s)\, dB_s\right)\right] = \int_0^T \mathbb{E}[f(s)g(s)]dt \tag{5}$$

as well as, with W_t as another independent Brownian motion, another corollary:

$$\mathbb{E}\left[W_t\left(\int_0^t g(s)\, dB_s\right)\right] = \int_0^t \mathbb{E}[g(s)]ds \tag{6}$$

Also, finally, using the independence property of the Brownian motions:

$$\mathbb{E}\left[\left(\int_0^T f(s,B_s)\, dB_s^j\right)\left(\int_0^T g(s,B_s)\, dB_s^i\right)\right] = 0 \tag{7}$$

when $i \neq j$.
We can now compute the first moment vector. Recall the SDE is given by:

$$\begin{aligned}
dX_s^1 &= \frac{1}{2}\left([\varsigma + \delta\cos(2k_B X_s^3)]\,dB_s^1 + \delta\sin(2k_B X_s^3)\,dB_s^2\right)\\
dX_s^2 &= \frac{1}{2}\left(\delta\sin(2k_B X_s^3)\,dB_s^1 + [\varsigma - \delta\cos(2k_B X_s^3)]\,dB_s^2\right)\\
dX_s^3 &= v_t\,dB_s^3
\end{aligned}$$

This can be rewritten in stochastic integral form via:

$$\begin{aligned}
2X_s^1 &= \int_0^t [\varsigma + \delta\cos(2k_B X_s^3)]\,dB_s^1 + \int_0^t \delta\sin(2k_B X_s^3)\,dB_s^2\\
&= \varsigma B_s^1 + \delta\int_0^t \cos(2k_B X_s^3)\,dB_s^1 + \delta\int_0^t \sin(2k_B X_s^3)\,dB_s^2\\
2X_s^2 &= \int_0^t \delta\sin(2k_B X_s^3)\,dB_s^1 + \int_0^t [\varsigma - \delta\cos(2k_B X_s^3)]\,dB_s^2\\
&= \varsigma B_s^2 + \delta\int_0^t \sin(2k_B X_s^3)\,dB_s^1 - \delta\int_0^t \cos(2k_B X_s^3)\,dB_s^2\\
X_s^3 &= v_t B_s^3
\end{aligned}$$

Using (1) and the fact that the integrals all vanish under expectation (because the integrand only ever depends on a Wiener process independent from the one in the differential), we get:

$$\mathbb{E}\left[X_t\right] = \vec{0}$$

This is intuitively similar to the fact that there is no drift for the empirical GHM.

Next, we can compute the second moments. Since the first moments are zero, the second moments directly correspond to the variance of the stochastic diffusion process.

The third component can be computed easily:

$$\mathbb{E}\left[(X_t^3)^2\right] = v_t^2\,\mathbb{E}\left[(B_t^3)^2\right] = v_t^2 t$$

Now for X_t^2, using $X_s^3 = v_t B_s^3$:

$$\begin{aligned}
4(X_t^2)^2 &= \left(\varsigma B_t^2 + \delta\int_0^t \sin(\phi)\,dB_s^1 - \delta\int_0^t \cos(\phi)\,dB_s^2\right)^2\\
&= \varsigma^2 (B_t^2)^2 + 2\varsigma\delta B_t^2\int_0^t \sin(\phi)\,dB_s^1\\
&\quad - 2\varsigma\delta B_t^2\int_0^t \cos(\phi)\,dB_s^2 - 2\delta^2\left(\int_0^t \sin(\phi)\,dB_s^1\right)\left(\int_0^t \cos(\phi)\,dB_s^2\right)\\
&\quad + \delta^2\left(\int_0^t \cos(\phi)\,dB_s^2\right)^2 + \delta^2\left(\int_0^t \sin(\phi)\,dB_s^1\right)^2
\end{aligned}$$

where we have defined $\phi = 2k_B v_t B_s^3$ for notational convenience. Now let us compute the result term-by-term.

$$\mathbb{E}\left[(v_f + v_t)^2 (B_t^2)^2\right] = (v_f + v_t)^2 t$$

By independence and (1):

$$\mathbb{E}\left[2\varsigma\delta B_t^2\int_0^t \sin(\phi)\,dB_s^1\right] = 2\varsigma\delta\mathbb{E}\left[B_t^2\right]\mathbb{E}\left[\int_0^t \sin(\phi)\,dB_s^1\right] = 0$$

Using (2), (5), and (6):

$$\mathbb{E}\left[-2\varsigma\delta B_t^2\int_0^t \cos(\phi)\,dB_s^2\right] = -2\varsigma\delta\int_0^t \mathbb{E}\left[\cos(\phi)\right]ds = \frac{-\varsigma\delta}{k_B^2 v_t^2}\left[1-\exp(-2k_B^2 v_t^2 t)\right]$$

Using (7):

$$\mathbb{E}\left[-2\delta^2\left(\int_0^t \sin(\phi)\,dB_s^1\right)\left(\int_0^t \cos(\phi)\,dB_s^2\right)\right] = 0$$

Using (3), (4), and $\cos^2(\theta) = 1-\sin^2(\theta)$:

$$\mathbb{E}\left[\delta^2\left(\int_0^t \sin(\phi)\,dB_s^1\right)^2\right] = \delta^2\int_0^t \mathbb{E}\left[\sin^2(\phi)\right]ds = \delta^2\left[\frac{t}{2} - \frac{1-\exp(-8k_B^2 v_t^2 t)}{16k_B^2 v_t^2}\right]$$

For the same reasons:

$$\mathbb{E}\left[\delta^2\left(\int_0^t \cos(\phi)\,dB_s^2\right)^2\right] = \delta^2\left[\frac{t}{2} + \frac{1-\exp(-8k_B^2 v_t^2 t)}{16k_B^2 v_t^2}\right]$$

Summing the expectations gives:

$$\mathbb{E}\left[(X_t^2)^2\right] = \frac{1}{4}\left[(\varsigma^2+\delta^2)t - \frac{\varsigma\delta}{k_B^2 v_t^2}\left[1-\exp(-2k_B^2 v_t^2 t)\right]\right]$$

The other dimensional component is almost the same:

$$\mathbb{E}\left[(X_t^1)^2\right] = \frac{1}{4}\left[(\varsigma^2+\delta^2)t + \frac{\varsigma\delta}{k_B^2 v_t^2}\left[1-\exp(-2k_B^2 v_t^2 t)\right]\right]$$

Given the first moment values, the variance is then:

$$\mathbb{V}[X_t] = t\begin{bmatrix}(v_f^2+v_t^2)/2\\(v_f^2+v_t^2)/2\\v_t^2\end{bmatrix} + \frac{\varsigma\delta\left[1-\exp(-2k_B^2 v_t^2 t)\right]}{4k_B^2 v_t^2}\begin{bmatrix}1\\-1\\0\end{bmatrix}$$

Note that, as $t \to 0$, $\mathbb{V}[X_t] \to 0$, i.e. the distribution converges to a Dirac delta function at $(0,0,0)$. This makes sense, as we assumed the diffusion starts at the origin with probability 1.

Similarly, with regards to the asymptotic variance as k_B shrinks to 0, we note that we get the following using L'Hôpital's rule:

$$\lim_{k_B\to 0}\frac{(v_f^2-v_t^2)\left[1-\exp(-2k_B^2 v_t^2 t)\right]}{4k_B^2 v_t^2} = -(v_f^2-v_t^2)(-t/2)$$

with which one can show that $\lim_{k_B\to 0}\mathbb{V}[X_t] = [v_f^2, v_t^2, v_t^2]^T t$.

Necessary Conditions for Variational Extrema

In this section we provide a proof for the transversality condition used in the analysis of diffusion bias and the Legendre condition used in the variational analysis of Ricci curvature.

We are concerned with variational functionals in n dimensions:

$$J(u) = \int_{\Omega} L\Big(u(x), Du(x)\Big)dx, \quad \text{or} \quad J(u) = \int_{\Omega} L\Big(u(x), Du(x), D^2u(x)\Big)dx$$

where $\Omega \subset \mathbb{R}^n$, $u : \Omega \to \mathbb{R}$, $Du = (\partial_{x_1}u, \ldots, \partial_{x_n}u)$ is the gradient of u, $D^2u = (\partial_{x_i x_j}u)_{i,j}$ its Hessian matrix, and dx denotes standard n-dimensional integration. Both u and L are expected to be suitably continuously differentiable. For clarity, we denote the independent variables of the integrand L by $z \in \mathbb{R}$, $p \in \mathbb{R}^n$, and, in the second order case, $q \in \mathbb{R}^{n^2}$ such that under the integral for $J(u)$ we have:

$$z = u(x), p = Du(x), q = D^2u(x)$$

In what follows by "minimizer" ("maximizer") we mean a local minimum (maximum) with respect to the weak norm given by $\|u\| = \sup_{\Omega}|u| + \sup_{\Omega}|Du|$ or in the second order case, $\|u\| = sup_{\Omega}|u| + \sup_{\Omega}|Du| + \sup_{\Omega}|D^2u|$.

First Order Conditions for Variational Extrema

Theorem 1. *Consider the variational integral*

$$J(u) = \int_{\Omega} L\big(u(x), Du(x)\big)dx$$

where $L(z,p) : \mathbb{R}\times\mathbb{R}^n \to \mathbb{R}$ is continuously differentiable and $\Omega \subset \mathbb{R}^n$ has smooth boundary. A necessary condition for $\bar{u}$ to be a weak local minimum of J is that the Euler-Lagrange equation $L_z - \operatorname{div}(L_p) = 0$ is satisfied for all $x \in \Omega$, where the LHS (referred to as $\delta J/\delta u$) is evaluated at $z = \bar{u}(x), p = D\bar{u}(x)$. Furthermore, if the value of u on $\partial\Omega$ is not constrained, the transversality condition $L_p \perp \hat{n}$ must also hold for all $x \in \partial\Omega$, where $\hat{n}$ is the normal vector to $\partial\Omega$.

Proof. We take as granted the fact that [3, pp 7]: *a necessary condition for $\bar{u}$ to be a weak local minimum of J is that the first variation $\delta J(\bar{u}, \varphi)$ vanishes for all admissible perturbations φ.* The difference between the constrained and unconstrained cases is reflected in the space of admissible perturbations: only in the constrained boundary case are we restricted to perturbations that vanish on the boundary, i.e $\varphi\Big|_{\partial\Omega} \equiv 0$. Expanding the first variation integral we get, for any admissible φ and any u:

$$\begin{aligned}\delta J(u,\varphi) = \frac{d}{ds}\Big|_{s=0} J(u+s\varphi) &= \frac{d}{ds}\Big|_{s=0}\int_{\Omega} L\big(u(x)+s\varphi(x), Du(x)+sD\varphi(x)\big)dx \\ &= \int_{\Omega}\frac{d}{ds}\Big|_{s=0} L\big(u+s\varphi, Du+sD\varphi\big)dx \\ &= \int_{\Omega}\Big[\varphi L_z + D\varphi\cdot L_p\Big]dx\end{aligned}$$

We now recall the identity $\operatorname{div}(\varphi L_p) = \varphi\operatorname{div}(Lp) + D\varphi\cdot L_p$ from multivariable calculus which together with an application of the divergence theorem gives:

$$(*) \qquad \delta J(u,\varphi) = \int_{\Omega}\varphi\Big[L_z - \operatorname{div}(L_p)\Big]dx + \int_{\partial\Omega}\varphi[L_p\cdot\hat{n}]dS$$

where dS denotes standard surface integration. We now note that in the constrained case the second integral in $(*)$ identically vanishes. However, perturbations that vanish on $\partial\Omega$ are admissible in both constrained and unconstrained cases. Therefore, in either case the first integral in $(*)$ must vanish for all φ that vanishes on $\partial\Omega$. The fundamental lemma [3, pp 16] now implies that the Euler-Lagrange equation must be satisfied for all $x \in \Omega$. In the unconstrained case, we additionally note that since the first intgeral in $(*)$ vanishes (by Euler-Lagrange) applying the fundamental lemma a second time to the second integral gives the transversality condition. □

Legendre Condition for Linearly Second Order Integrands

We take the following standard result as granted [3, pp 223]: *a necessary condition for $\bar{u}$ to be a weak local minimizer (maximizer) of J is that for all admissible perturbations φ we have*

$$\delta^2 J(\bar{u},\varphi) = \frac{d^2}{ds^2}\Big|_{s=0} J(\bar{u}+s\varphi) \geq 0 \qquad (\leq 0)$$

Theorem 2. *Consider the variational functional in n dimensions:*

$$J(u) = \int_{\Omega} L\left(u(x), Du(x), D^2u(x)\right)dx$$

If L is such that $L_{qq} \equiv L_{pq} \equiv L_{zq} \equiv 0$ over Ω then a necessary condition for $\bar{u}$ to be a weak local minimizer (maximizer) for J is that the $n \times n$ matrix $L_{pp}\Big|_{x,\bar{u}}$ is positive (negative) semidefinite, $L_{pp} \succeq 0$ ($L_{pp} \preceq 0$), for all $x \in \Omega$.

Proof. We prove the result for minimizers. The proof for the maximizer case follows by applying the same argument to

$$-J(u) = \int_\Omega -L(u(x), Du(x), D^2u(x))dx$$

We first expand the second variation integral:

$$\begin{aligned}
\delta^2 J(\bar{u},\varphi) = \frac{d^2}{ds^2}\Big|_{s=0} J(u+s\varphi) &= \frac{d^2}{ds^2}\Big|_{s=0} \int_\Omega L\left(u(x)+s\varphi(x), Du(x)+sD\varphi(x), D^2u(x)+sD^2\varphi(x)\right)dx \\
&= \int_\Omega \frac{d^2}{ds^2}\Big|_{s=0} L\left(u(x)+s\varphi(x), Du(x)+sD\varphi(x), D^2u(x)+sD^2\varphi(x)\right)dx \\
&= \int_\Omega \varphi^2 L_{zz} + \sum_i \partial_{x_i}\varphi L_{zp_i} + \sum_{i,j} \partial_{x_i x_j}\varphi L_{zq_{ij}} \\
&\quad + \int_\Omega \sum_k \partial_{x_k}\varphi \left[\varphi L_{zp_k} + \sum_i \partial_{x_i} L_{p_k p_i} + \sum_{i,j} \partial_{x_i x_j}\varphi L_{p_k q_{ij}}\right] \\
&\quad + \int_\Omega \sum_{k,l} \partial_{x_k x_l}\varphi \left[\varphi L_{q_{kl} z} + \sum_i \partial_{x_i}\varphi L_{q_{kl} p_i} + \sum_{i,j} \partial_{x_i x_j}\varphi L_{q_{kl} q_{ij}}\right] \\
(*) &= \int_\Omega \left[\varphi^2 L_{zz} + 2\varphi L_{zp}\cdot D\varphi + D\varphi^\top L_{pp} D\varphi\right] dx
\end{aligned}$$

where all sums run over indices $1,\ldots,n$ and we have used the fact that $L_{qq} \equiv L_{pq} \equiv L_{zq} \equiv 0$.

The key idea now is that the last term in $(*)$ is dominant in the sense that we can find custom perturbations (to be specified) φ_N such that

$$\left|D\varphi_N^\top L_{pp} D\varphi_N\right| \to \infty \quad \text{as} \quad N \to \infty$$

while $\left|\varphi_N^2 L_{zz}\right|$ and $\left|\varphi_N L_{zp}\cdot D\varphi_N\right|$ remain bounded. From this we will argue that if $L_{pp}\Big|_{x,\bar{u}} \succeq 0$ is violated at any x we can create perturbations that drive the dominant term to $-\infty$ hence making the second variation negative contradicting the standard necessary condition.

Suppose $L_{pp}\Big|_{x_\circ,\bar{u}} \not\succeq 0$ for some $x_\circ$; fix a negative eigenvalue $-\lambda$ with a unit eigenvector ϕ. This means:

$$\phi^\top L_{pp}\Big|_{x_\circ,\bar{u}}\phi = -\lambda < 0$$

which by continuity of L_{pp} implies that for over some open ball $B := B(x_\circ, r)$ we have

$$\phi^\top L_{pp}\Big|_{x,\bar{u}}\phi < -\frac{\lambda}{2} < 0$$

We now consider the perturbations

$$\varphi_N(x) := f(N)\sin(N\phi\cdot x)\mathbf{1}_B(x)$$

where $\mathbf{1}$ is the indicator function, i.e $\text{supp}\varphi_N \subset B$, and $f(N) > 0$ is to be determined. We note that for any vector a we have $D\sin(a\cdot x) = \cos(a\cdot x)a$. Thus

$$\begin{aligned}
\delta^2 J(\bar{u},\varphi_N) &= \int_B f(N)^2 \sin^2(\phi\cdot x) L_{zz}\Big|_{x,\bar{u}} dx \\
&\quad + \int_B N f(N)^2 \sin(2\phi\cdot x)\phi\cdot L_{zp}\Big|_{x,\bar{u}} dx \\
&\quad + \int_B N^2 f(N)^2 \cos^2(\phi\cdot x)\phi^\top L_{pp}\Big|_{x,\bar{u}}\phi \\
&\le C_1\left[f(N)^2 + N f(N)^2\right] - C_2 N^2 f(N)^2
\end{aligned}$$

where $C_1 = \mathrm{vol}(B)\max\left\{\sup_B |L_{zz}|, \sup_B |L_{zp}|\right\}$ and $C_2 = \mathrm{vol}(B)\lambda/2$. By choosing $f(N) = N^{-3/4}$ we get

$$\delta^2 J(\bar{u}, \varphi_N) \le C_1 \left[\frac{1}{N\sqrt{N}} + \frac{1}{\sqrt{N}}\right] - C_2\sqrt{N}$$

The first term on the right hand side vanishes as $N \to \infty$ while the second term goes to $-\infty$, that is, for large enough N we get $\delta^2 J(\bar{u}, \varphi_N) < 0$. □

References

1. Young, R. J. & Panfilov, A. V. Anisotropy of wave propagation in the heart can be modeled by a Riemannian electrophysiological metric. *Proc. Natl. Acad. Sci.* **107**, 15063–15068 (2010).

2. Savadjiev, P. *et al.* Heart wall myofibers are arranged in minimal surfaces to optimize organ function. *Proc. Natl. Acad. Sci.* **109**, 9248–9253 (2012).

3. Giaquinta, M. & Hildebrandt, S. *Calculus of variations I* (Springer-Verlag, 2004).
